# The Use of Iron Oxide Nanoparticles to Reprogram Macrophage Responses and the Immunological Tumor Microenvironment

**DOI:** 10.3389/fimmu.2021.693709

**Published:** 2021-06-09

**Authors:** Vladimir Mulens-Arias, José Manuel Rojas, Domingo F. Barber

**Affiliations:** ^1^ Department of Immunology and Oncology, and NanoBiomedicine Initiative, Centro Nacional de Biotecnología (CNB)-CSIC, Madrid, Spain; ^2^ Centro de Investigación en Sanidad Animal, Centro Nacional Instituto de Investigación y Tecnología Agraria y Alimentaria (CISA-INIA)-CSIC, Valdeolmos, Madrid, Spain

**Keywords:** iron oxide nanoparticles, nanoparticle–macrophage interaction, macrophage polarization, tumor associated macrophages, therapeutic applications

## Abstract

The synthesis and functionalization of iron oxide nanoparticles (IONPs) is versatile, which has enhanced the interest in studying them as theranostic agents over recent years. As IONPs begin to be used for different biomedical applications, it is important to know how they affect the immune system and its different cell types, especially their interaction with the macrophages that are involved in their clearance. How immune cells respond to therapeutic interventions can condition the systemic and local tissue response, and hence, the final therapeutic outcome. Thus, it is fundamental to understand the effects that IONPs have on the immune response, especially in cancer immunotherapy. The biological effects of IONPs may be the result of intrinsic features of their iron oxide core, inducing reactive oxygen species (ROS) and modulating intracellular redox and iron metabolism. Alternatively, their effects are driven by the nanoparticle coating, for example, through cell membrane receptor engagement. Indeed, exploiting these properties of IONPs could lead to the development of innovative therapies. In this review, after a presentation of the elements that make up the tumor immunological microenvironment, we will review and discuss what is currently known about the immunomodulatory mechanisms triggered by IONPs, mainly focusing on macrophage polarization and reprogramming. Consequently, we will discuss the implications of these findings in the context of plausible therapeutic scenarios for cancer immunotherapy.

## Introduction

The highly innovative field of nanotheranostics has been expanding now for more than two decades, with easy-to-scale nanomaterials emerging as potential candidates to treat a variety of pathologies, such as cancer (1–4), autoimmune diseases (5, 6) or neurodegenerative disorders (7, 8). The therapeutic interest in nanomaterials, and particularly in nanoparticles, is in part kindled by the chemical and physical versatility of these materials. Nanoparticles can be functionalized with targeting moieties (9) or drugs (10), and their surface can be built for specific biomolecule release using molecular domains responsive to stimuli like pH (11, 12) or reactive oxygen species (ROS (13, 14). In addition, they also possess physical properties associated with their core that can be exploited, such as magnetism (15) and plasmon coupling (16).

Iron oxide nanoparticles (IONPs) are of particular therapeutic interest due to their magnetic properties and their flexibility for surface functionalization. IONPs have been used as contrast agents and as heat-inducers through the application of an external magnetic field (17, 18). Their versatility in terms of surface functionalization means they can target diverse molecules and they can be used to ensure the correct localized delivery of different cargos, such as drugs, RNAs, cytokines or antibodies (15). Importantly, IONPs also exhibit intrinsic biological activity in cellular systems, including the immune system, which can be exploited to broaden their therapeutic potential. This review will first outline the main characteristics of the tumor microenvironment (TME), emphasizing the influence of tumor-associated macrophages (TAMs), and subsequently, we will address the impact that IONPs have on macrophage reprogramming and the implications of this for cancer immunotherapy.

## Immunological Tumor Microenvironment

**Figure 1:**
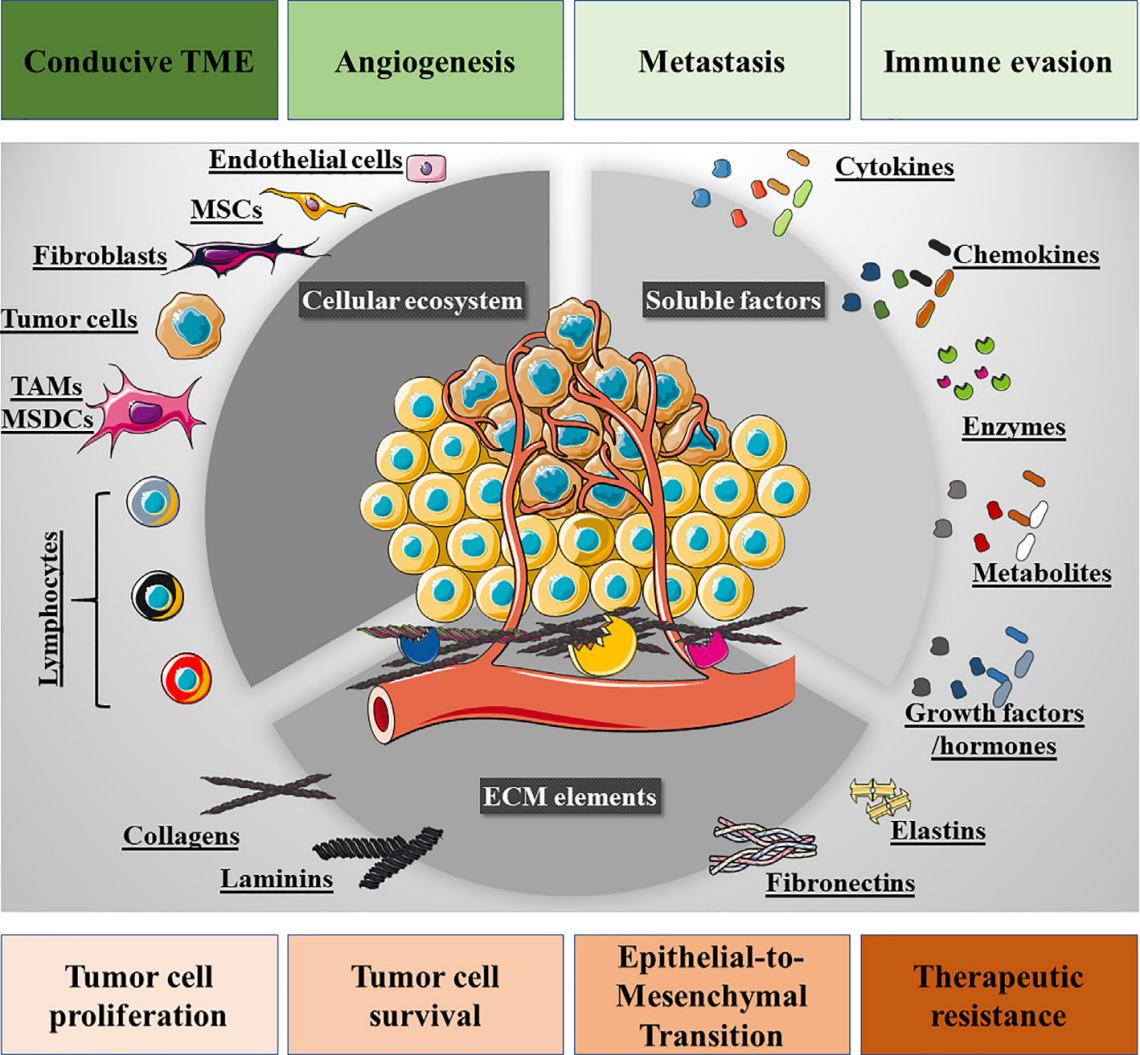
Overview of the tumor microenvironment (TME). Three ecosystems contribute to the TME: firstly, the cellular ecosystem that is composed of immune cells (lymphoid and myeloid), fibroblasts, mesenchymal stem cells (MSCs), pericytes, endothelial cells, and tumor cells. Secondly, the cell-to-cell membrane interactions and soluble secreted factors that participate in the intricate interplay among these cells, *e.g.*, cytokines, chemokines, growth factors, hormones, proteolytic enzymes, and metabolites. Thirdly, the extracellular matrix (ECM) bed on which the cellular ecosystem resides, also providing biological signals to the tumor and stromal cells through ECM-derived peptides and the structural domains of its proteins. The interplay of these signaling networks and ecosystems promotes tumor cell proliferation, survival, epithelial-to-mesenchymal transition, drug resistance and loco-regional modulation, such that the TME is conducive to tumor cell invasion and metastatic spreading, angiogenesis and immune cell evasion.

Cancer is a complex and heterogeneous disease that involves the dysregulation of various cell processes, such as metabolism (19), proliferation (20), intracellular pH dynamics (21), redox signaling (22), and migration/invasion (23, 24). The complexity of this disease is also reflected by the different ecosystems that constitute a permissive TME (25, 26). A close inspection of the TME reveals a network of cellular and non-cellular components that provide the signals that control tumor cell survival, proliferation, angiogenesis, immune evasion and metastasis. We can divide the TME landscape into three ecosystems: 1) the cellular compartment; 2) the soluble factors; and 3) the extracellular matrix (ECM: **Figure 1** and **Table 1**). The tumor niche is a very dynamic 3D structure in which stromal cells play a crucial role in regulating different stages of tumor development and in which there is also an intricate interplay among these cells. The TME cell ecosystem also includes a plethora of non-immune stromal cell types, such as cancer-associated fibroblasts (CAFs (54), mesenchymal stem cells (MSCs), pericytes, adipocytes, endothelial and vascular cells. Notably, these cells exhibit a high degree of plasticity and they may originate through trans-differentiation. For instance, breast cancer CAFs may stem from resident fibroblasts, from breast epithelial cells *via* the epithelial-to-mesenchymal transition (EMT) or from pericytes in the perivascular niche (55, 56). CAFs may also be derived from bone marrow-derived mesenchymal stem cells (BM-MSCs), as PDGFR-α^−^, CD45^−^, CD34^−^ BM-MSCs are recruited into primary breast tumors to differentiate into α-SMA^+^, PDGFR-α^−^, CD45^−^, CD34^−^ CAFs (57). This fact highlights the complex transcriptional reprogramming that many stromal cells go through, suggesting that the cellular ecosystem in the TME is in constant transcriptional flux (58, 59). Indeed, this dynamic transcriptional program is likely to constantly redefine the immunological landscape of the TME.

**Table 1:** Examples of TME ecosystems and their implications in the progression of three significant cancers: breast, lung and colorectal.

Tumor	Component	Implications
Breast tumors	Cancer-associated fibroblasts (CAFs)	Tumor invasion through stromatogenesis (27)
		Tumor EMT through TGF-β1 (28, 29)
		Self-renewal of breast cancer stem cells (30)
		Tumor progression through growth factors, e.g., SDF-1 (31), FGF-β (32)
		Tumor progression through cytokines and chemokines, e.g., CXCL14 (33), CXCL16 (34), IL-4 & IL-6 (35), IL-33 (36)
Breast tumors	Mesenchymal stem cells (MSCs)	Immunosuppression through the CCL5/PD-L1 axis (37)
		Enhanced tumor progression through CCL5 and IL-6 (38)
Lung tumors	CAFs	Chemoresistance through upregulation of TNFSF4 (39) and/or ANXA3 (40)
		Immunosuppression by modulating TIM (41)
		Enhanced growth and invasion through VCAM-1 secretion (42) and induction of PD-L1 (43)
Colorectal cancers	CAFs	Enhanced metastasis through HGF (44)
		Chemoresistance through exosomal lncRNA H19 (45)
		Enhanced tumor cell migration/invasion through Wnt2 (46), IL-33 (47), CLEC3B (48) and/or SNAIL-1 (49)
Colorectal cancers	Pericytes	Enhanced tumor cell invasion through the TGF-β1/IGFBP-3 axis (50)
Colorectal cancers	MSCs	Enhanced tumor progression through IL-8 (51), TGF-β1/CXCR4 (52), CCL5/β-catetin/Slug (53)

The TME is also comprised of tumor-infiltrating immune cells, both innate immune cells (monocytes, macrophages, and NK cells) and adaptive immune cells (T and B cells), that define the tumor immune microenvironment (TIME). Dynamic communication takes place within this ecosystem that are mediated by cell-to-cell contacts and cell-derived soluble factors. The intermediates derived from stromal and tumor cells, such as cytokines, chemokines, and ROS, promote immune evasion by inducing CD8^+^ T cell anergy/exhaustion, T regulatory (Tregs) cells, suppressor dendritic cells (DCs), and M2 macrophage differentiation (60). As a result, tumors escape immune surveillance and they adopt a metastatic phenotype through modulation of the EMT, enhanced angiogenesis and ECM degradation.

The non-cellular TME network is comprised of ECM components [*e.g.*, collagens (61), fibronectin (62), elastin (63), and laminin (64)], and soluble cellular derivatives [*e.g.*, cytokines, chemokines (65), hormones (66), metabolites (67, 68) and growth factors (69)]. This non-cellular network is responsible for cell-to-cell crosstalk, ultimately shaping the pro-malignant environment.

**Figure 2:**
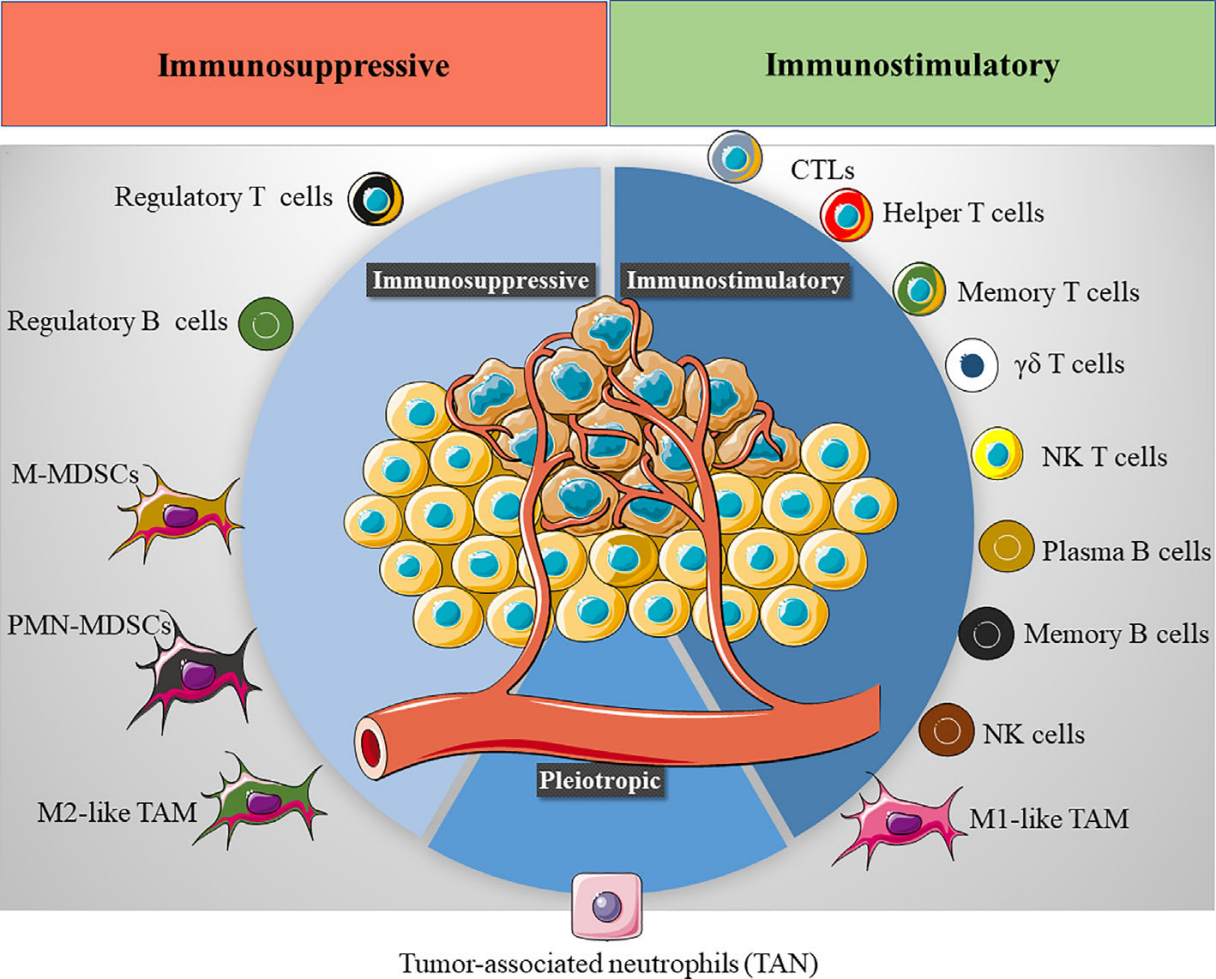
The tumor immune microenvironment (TIME). Several immune cells are found in the TIME, exhibiting either an immunostimulatory (CTLs, cytotoxic T cells, helper T cells, memory T cells, γδ T cells, NK T cells, plasma B cells, memory B cells, NK cells and M1-like TAMs) or immunosuppressive phenotype (Tregs cells, regulatory B cells, M-MDSCs, monocytic monocyte-derived suppressor cells, PMN-MDSCs, polymorphonuclear monocyte-derived suppressor cells and M2-like TAMs). The final immunological response in the TME will depend on the balance between these immunomodulatory populations.

However, the immunological landscape within the TME has emerged as a crucial variable for cancer progression and treatment, and understanding the TIME has become a critical step in designing efficient immunotherapies for cancer. Indeed, the TIME defines the prognosis of cancer patients (70, 71) and their therapeutic response to immunotherapies like checkpoint inhibitors (72, 73), T-cell transfer (74), or therapeutic vaccines (75). Driven by tumor cell plasticity, the TIME is a dynamic system where diverse innate and adaptive immune cells co-exist, continually changing over time in response to the reprogramming of tumor cell transcription (**Figure 2**). To better comprehend the TIME’s influence on cancer prognosis, the TIME can be divided into the T cell-inflammatory microenvironment and non-T cell-inflammatory microenvironment. The first of these is characterized by the infiltration of T cell subsets and macrophages, whereas the second is mainly composed of TAMs. Of all immune cells, TAMs play a pivotal role in defining the tumor immunological landscape and thus, they have been the target of various therapeutic approaches.

### Immunosuppressive Tumor-Associated Macrophages

**Figure 3:**
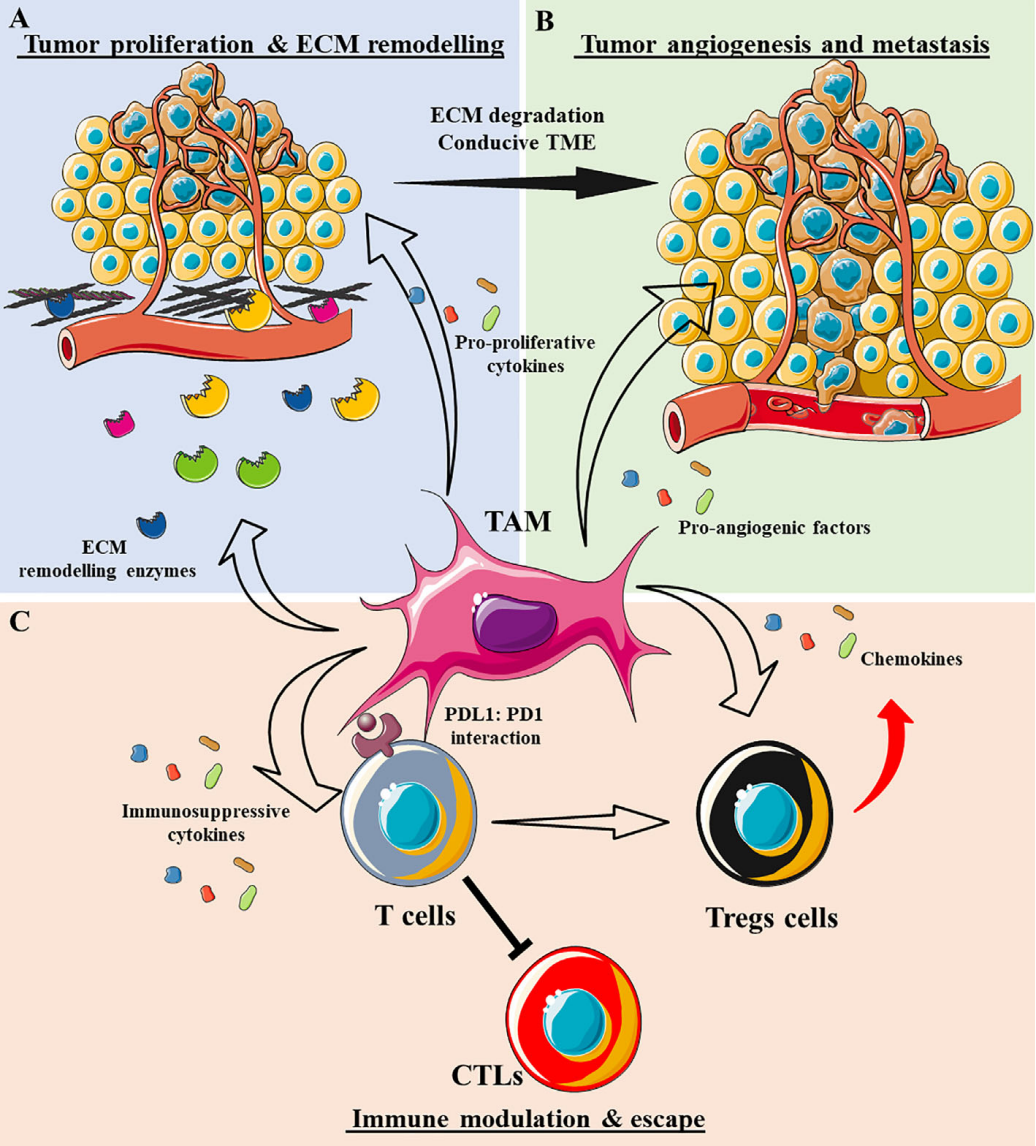
The role of tumor-associated macrophages (TAMs) in shaping the tumor microenvironment (TME). **(A)** TAMs secrete a plethora of enzymes that degrade ECM components, such as metalloproteinases (MMPs), cathepsins, disintegrin and metalloproteinase (ADAM)-family proteases, and tissue inhibitors of metalloproteinases (TIMPs). As a result, the ECM becomes destructured and conducive to tumor cell invasion. TAMs also secrete cytokines that support tumor cell proliferation, e.g., TGF-β1, IL-10, IL-6, IL-1β, and EGF. **(B)** TAMs secrete various pro-angiogenic factors that induce vessel formation, *e.g.*, VEGF-A, bFGF, IL-6, and TNFα. Together with ECM degradation, tumor angiogenesis permits the systemic dissemination of tumor cells. **(C)** TAMs adopt an immunosuppressive phenotype by secreting many anti-inflammatory cytokines/chemokines, *e.g.*, IL-10, TGF-β1, CCL17, CCL18, and CCL22, inhibiting cytotoxic T cells (CTLs) and attracting or differentiating T cells into regulatory T cells. TAMs can also exhaust CTLs by direct engagement of anti-inflammatory cognates receptors like PD1-PD-L1.

TAMs are tumor-enriched immunosuppressor cells that exert a pivotal influence on tumor progression and metastasis. Since their first description 30 years ago (76), TAMs have been characterized as potent pro-tumorigenic agents that act primarily by modulating the natural (and induced) anti-tumor response, ECM remodeling, and inducing angiogenesis, not only leading to tumor cell survival and proliferation but also, to their dissemination (**Figure 3**). It is currently accepted that the TAM phenotype resembles the alternatively activated macrophage M2 phenotype (Arginase 1^+^, CD163^+^, CD206^+^, CD209^+^, FIZZ1^+,^ and Ym1/2^+^), which can be subdivided into four subtypes: M2a, M2b, M2c, and M2d (77). These subtypes are generated by the stimuli triggering macrophage differentiation and some specific phenotypic markers (**Table 2**). However, it is generally accepted that TAMs retain a high degree of plasticity, permitting several different subtypes to co-exist simultaneously and their trans-differentiation into each different subtype depending on the TME signals available.

**Table 2:** **|** M2 macrophage subtypes and their involvement in tumor development.

M2 Subtype	Stimuli	Phenotype	Functions
M2a	IL-4/IL13	IL-10, TGF-β1, IL-1R agonist	To promote a Th2 response and tumor cell invasiveness (78, 79)
M2b	IL-1β, immune complexes and LPS	IL-1, IL-6, IL-10, TNFα	Pro-Th2 activity, tumor progression and immunotherapy resistance (80)
M2c	IL-10, TGF-β1, glucocorticoids	IL-10, TGF-β1	ECM remodeling and to promote tumor migration/invasion (81, 82)
M2d	IL-6, adenosine	IL-10, IL-12, TNFα, TGF-β1	Tumor progression and invasion (83)

In general, blood monocytes infiltrate the TME, and along with the tumor-resident macrophages, they represent a source of TAMs. In this context, tumor cells shape the macrophage’s immunosuppressive phenotype by secreting anti-inflammatory interleukins and other metabolites. The TAMs then inhibit tumor-infiltrating T cells directly through receptor-ligand cognates [*e.g.*, PD-1:PD-L1 (84)] or by releasing anti-inflammatory cytokines (IL-10, TGF-β1, and IL-6). Concomitantly, the TAMs can produce different proteolytic enzymes such as metalloproteinases (MMPs), cathepsins, and disintegrin and metalloproteinase-like proteases (ADAMs), thereby producing a profound ECM remodeling. Consequently, the ECM becomes conducive to invasion, and it facilitates tumor cell dissemination into the surrounding tissue and peripheral circulation. TAMs can further enhance tumor invasiveness by inducing angiogenesis, mediated by various cytokines and growth factors like VEGF-A (85) and IL-8 (86). Since TAMs are involved in tumor progression, the induction of a specific phenotype that switches these cells towards a pro-immunogenic profile has been proposed as an attractive therapeutic tool to enhance local anti-tumor immune responses.

The modulation of TAM activity is a plausible and promising therapeutic approach to combat tumors when combined with cancer immunotherapies. Indeed, multiple drugs that modulate the pro-tumor activity of TAMs have been tested, including bisphosphonates (87) and zoledronic acid (88) in particular, or chemotherapeutic drugs like docetaxel and cyclophosphamide (89). While zoledronic acid can revert the M2 TAM phenotype in breast tumors into an M1-like phenotype or induce TAM apoptosis, the chemotherapeutic drugs can promote an M1-phenotype that secretes pro-inflammatory cytokines like IL-12, thus driving an anti-tumor effect. In this context, nanoparticles that modulate TAM activity, particularly IONPs, provide new and innovative tools to prolong anti-tumor responses *in situ*.

## Intrinsic Modulation of the TIME by Iron Oxide Nanoparticles (IONPs)

IONPs have been studied extensively as an effective magnetic nanocarrier for various cargos, such as drugs (15), cytokines (90, 91), siRNAs (92), and adjuvants (93). There are several motives for the increasing interest in IONPs as nanocarriers. First, the IONP core responds to an external electromagnetic field that permits their use in applications like magnetic targeting, magnetic resonance imaging (MRI) or the induction of local hyperthermia. Second, mammalian cells have efficient iron metabolism that can prevent the cells from suffering iron-related toxicity. Third, the IONP surface provides a chemical interface that can be easily modified with a number of polymers and moieties, which when combined with the high surface-to-volume ratio, facilitate the delivery of wide range of cargoes.

However, IONPs also produce interesting intrinsic biological effects that provide added therapeutic benefits to IONP-based nanomedicines. We demonstrated that polyethyleneimine (PEI)-coated IONPs can inhibit the migration and invasion of tumor cells (94), and impair angiogenesis (95). More importantly, the intrinsic biological effects of IONPs arise from their surface coating and the surrounding protein corona, as well as the free intracellular iron derived from IONP degradation. While IONP surface microdomains are primarily involved in the nanoparticle’s interaction with cell membrane receptors, soluble factors, and intracellular components, the released intracellular iron actively changes the intracellular redox status through the Fenton reaction (96), modulating several iron-regulated genes. Since macrophages contribute to the TIME, their interaction with IONPs can define the theranostic outcome and provide an invaluable tool to reprogram the phenotype of TAMs. The most recent findings on how IONPs affect macrophage activation are summarized in **Table 3**.

**Table 3:** **|** Example of the effects of iron oxide nanoparticles on macrophage polarization.

Iron oxide nanoparticles	Cell model	Mechanisms	Effects exerted
PLGA@Fe_3_O_4_ & CD206-Ab-PLGA@Fe_3_O_4_ (97)	*In vitro:* IL-4-stimulated RAW 264.7 cells	ROS production	↑TNFα, iNOS, IL-1β
	*In vivo:* tumor model 4T1		↓Arg1, IL-10, TGF-β1
			↑CD86^+^ (M1) TAMs *in vivo*
Negative charged SPION	*In vitro:* RAW 264.7 cells	ROS production	↑TNFα, iNOS
Neutral charged SPION (PEG-coated)	*In vivo:* tumor model HT1080		↓IL-10, VEGF
			↓Tumor growth
Positive charged SPION (98)			
Ferumoxytol (Feraheme™) (99)	*In vitro*: Co-culture RAW 264.7/MMTV-PyMT tumor cells	Tumor cell apoptosis	↑Pro-M1 genes (*TNFA, INOS, CD86, ARG1*)
	*In vivo: tumor model MMTV-PyMT*		↓Pro-M2 genes (*IL10, CD206*)
	*In vivo metastasis: tumor model KP1*		↓Tumor growth and lung/liver metastases-*in vivo*
			↑M1 macrophage polarization *in vivo*
4-nm amphiphilic (PMA)@Fe_3_O_4_ (100)	*In vitro:* RAW 264.7 cells	Vacuolization, lysosomal damage	↑Pro-M1 genes (*TNFA, CD86, NFKB*)
			↓Pro-M2 genes (*CD206*)
Polyethyleneimine@Fe_3_O_4_ (101)	*In vitro:* RAW 264.7 cells, mouse peritoneal macrophages, THP1 cells	TLR4 activation, ROS production	↑IL-12, IL-10, CD80, CD86, CD40, I-A/I-E
			↑MAPK activation
Resovist™ & Ferumoxytol (Feraheme™) (102)	*In vitro:* Bone marrow-derived macrophages (BMDMs)	TLR4 activation	↑Pro-inflammatory factors
	*In vivo*: liver		↑Autophagy
DMSA@SPION, APS@SPION, & AD@SPION (103)	*In vitro:*	ROS production	↑IL-10
	M2 Macrophages: IL-4-*stimulated* Bone marrow-derived macrophages (BMDMs) and PMA-stimulated THP1		↑MAPK activation
			↑Cell invasion
			↓Cell migration
Resovist™ (104)	*In vitro:* M2 Macrophages: IL-4/IL-13-*stimulated* PMA-differentiated THP1		Induce a shift towards a M1 phenotype ↑CD86, TNF-α, Ferritin, Cathepsin L
2-kDa PEG@SPIONs (105)	*In vitro:* LPS-stimulated RAW 264.7 cells	Inhibition of TLR4 signaling	↓IL-6, TNFα, iNOS
100 nm large maghemite (Fe_2_O_3_) nanoparticles (106)	*In vitro:* J774A.1 cells	Iron uptake & Fenton reactions	↓Phagocytic rate
			↓LPS-dependent response

To understand how IONPs affect macrophage polarization, we have to consider the internalization process as at least three different steps, during which IONPs can engage with different signaling cascades: 1) IONP interaction with the cell membrane; 2) endocytosis and endolysosomal trafficking; and 3) IONP degradation. In each step the IONPs are exposed to diverse biological milieu and ultimately, this determines the indirect or direct engagement that drives macrophage transcriptional reprogramming and shifts in phenotype. This effect on transcriptional reprogramming of macrophages has been assessed by several groups whereby key transcription factors such as STAT family (107) and c-Fos/c-Jun complex (107) are upregulated upon IONP treatment. Noteworthy, IONPs appear to induce a variety of transcription factors related to MAPK pathways and the innate response, including the TLR-AP-1 signaling pathway (108). This complex reprogramming was revealed by Liu Y et al., who observed that the DMSA-coated IONPs engaged the activation of the signaling pathways mentioned above (107). Therefore, the IONPs can trigger a multifactorial transcription reprogramming of macrophages where several signaling pathways are involved.

It is important to note that among the transcriptional reprogramming that IONPs can induce in macrophages, some are related to cell death processes such as apoptosis, ferroptosis, and autophagy. The balance between all signaling pathways activated by a particular IONPs will determine the macrophage fate. In this review, we focus on the transcriptional reprogramming of macrophage response in terms of the immune response and suggest other comprehensive and recent studies on the toxicity of IONPs that can be more thorough in this sense (109, 110).

The coating of IONPs influences their interaction with cell membrane-associated proteins like receptors, thereby triggering signaling cascades that can activate macrophages. As such, IONPs with a positively charged coating consistently polarize macrophages towards a M1-like phenotype. Indeed, when macrophages are treated with PEI-coated IONPs, a straightforward program of M1 activation occurs, enhancing co-stimulatory receptors like CD40, CD80, and CD86, along with the secretion of the pro-inflammatory cytokine, IL-12 (101). When analyzing the transcriptional reprogramming induced by PEI-coated IONPs, several pro-inflammatory genes were seen to be upregulated (i.e., *Il1b*, *Tnfa*, *Ccl2* and *Il6*). However, the most exciting finding was the involvement of the toll-like receptor 4 (TLR-4) in PEI-coated IONP-induced macrophage activation (101). The PEI polymer appears to engage TLR-4 activation, stimulating the mitogen-activating protein kinase (MAPK). Two commercially available IONPs (carboxydextran-coated Resovist and carboxylmethyl-dextran coated feraheme) have also been demonstrated to induce macrophage activation through TLR-4 engagement, indicating that different IONP coatings can activate macrophages in this way, although activation by these IONPs induces autophagy (102). Other effects of IONPs were at least partly associated with different TLRs, including the cell membrane TLR2, TLR4, and TLR6, and the intracellular TLR8. Indeed, IONP size influences TLR activation as a relatively small IONP (10 nm) can enhance TLR2, TLR6, TLR4, and TLR8-induced cytokine secretion in peripheral blood, whereas a larger IONP (30 nm) only affects TLR2 and TLR6-dependent cytokine secretion (108). Although a direct interaction between the IONPs and the cell surface TLRs has yet to be demonstrated, the dependence of cytokine enhancement on the formation of a complex between TLR4/MD2 and the CD14 co-receptor suggests that a physical interaction between the TLR4 complex and IONPs could be responsible for the increase in TLR4 activity. However, elsewhere IONPs were shown to interfere with TLR4 agonist activation, suggesting that this mechanism could depend on the type of IONP (111).

In addition, it has been shown that IONPs with opposite surface charges promote similar macrophage repolarization. Two opposite charged IONPs induced an M1-like phenotype in RAW 264.7 macrophages, although negatively charged IONPs appeared to be more potent in promoting this effect (98) and neutral IONPs have a negligible impact. The crucial role that such M1-differentiated macrophages can play within the TIME was also addressed and there was significant tumor growth retardation when IONP^+^ or IONP^-^ treated macrophages were co-inoculated with HT1080 human fibrosarcoma cells, reflecting the anti-tumor effect of these repolarized M1-like macrophages (98).

IONP morphology also plays a critical role in determining the degree of macrophage activation. Using IONPs with four distinct morphologies (octopod, plate, cube, and spherical), yet with a comparable aspect ratios and surface charge, the IONPs with an octopod or plate morphology were seen to significantly activate the inflammasome, as measured by IL-1β secretion (112). More importantly, this dependence on morphology appeared to be related to the nanoparticle’s capacity to induce ROS production. IONP size also affected the extent of inflammasome activation in macrophages, with spherical IONPs of ~30 nm inducing significantly more IL-1β release than larger spherical IONPs of ~80 and 120 nm (113). ROS production appears to be a common molecular mechanism for the effect of IONPs on macrophage activation, although this result also seems to depend on lysosomal destabilization and may reflect another common phenomenon. The involvement of ROS in IONP-induced macrophage activation is related to the central role these metabolites play in macrophage cell biology as short-lived second messengers. ROS mediate the oxidation of thiol groups in several proteins, altering their structure and hence, their function. The MAPK pathway is ROS-sensitive and it regulates several biological processes like cell proliferation, apoptosis, and the innate immune response. In this regard, ROS have been implicated in the induction and maintenance of an M1-like status of macrophages through the activation of NFκB and p38 MAPK signaling. In the former situation, ROS trigger the phosphorylation of the NFκB inhibitor, IκB, thereby activating NFκB (114). In the latter, ROS induce the phosphorylation of the apoptosis signaling-regulating kinase 1 (ASK1) and the downstream activation of the p38 MAPK (115). However, ROS can either activate or inhibit NFκB in a context-dependent manner, highlighting the need to characterize the effect of IONP-triggered ROS production on NFκB activation in a cell-type and context-dependent manner (116). In addition to MAPK, the phosphoinositide-3 kinase (PI3K) is also regulated by ROS, sensitizing macrophages to hormone, cytokine, and growth factor signaling (117).

IONP phagocytosis can lead to autophagy, as is the case for the two FDA-approved IONPs, resovist and ferumoxytol that induce the appearance of an early autophagic vacuole and eventually, IONPs-containing double-membrane autophagic vacuoles, small internal vesicles, and cellular and membrane debris (102). These events were accompanied by the accumulation of LC3 puncta and overexpression of the p62/SQSTM1-positive sequestosome (118–120). In accordance with the involvement of TLRs in this effect, the TLR4-p38-Nrf2 pathway appears to mediate IONP-induced autophagy as opposed to the classic autophagy machinery dependent on ATG5/12. Indeed, pre-treatment with the TLR4 signaling inhibitor, CLI-095, prevented IONP-loaded macrophages from inducing the aforementioned structural changes (102).

**Figure 4:**
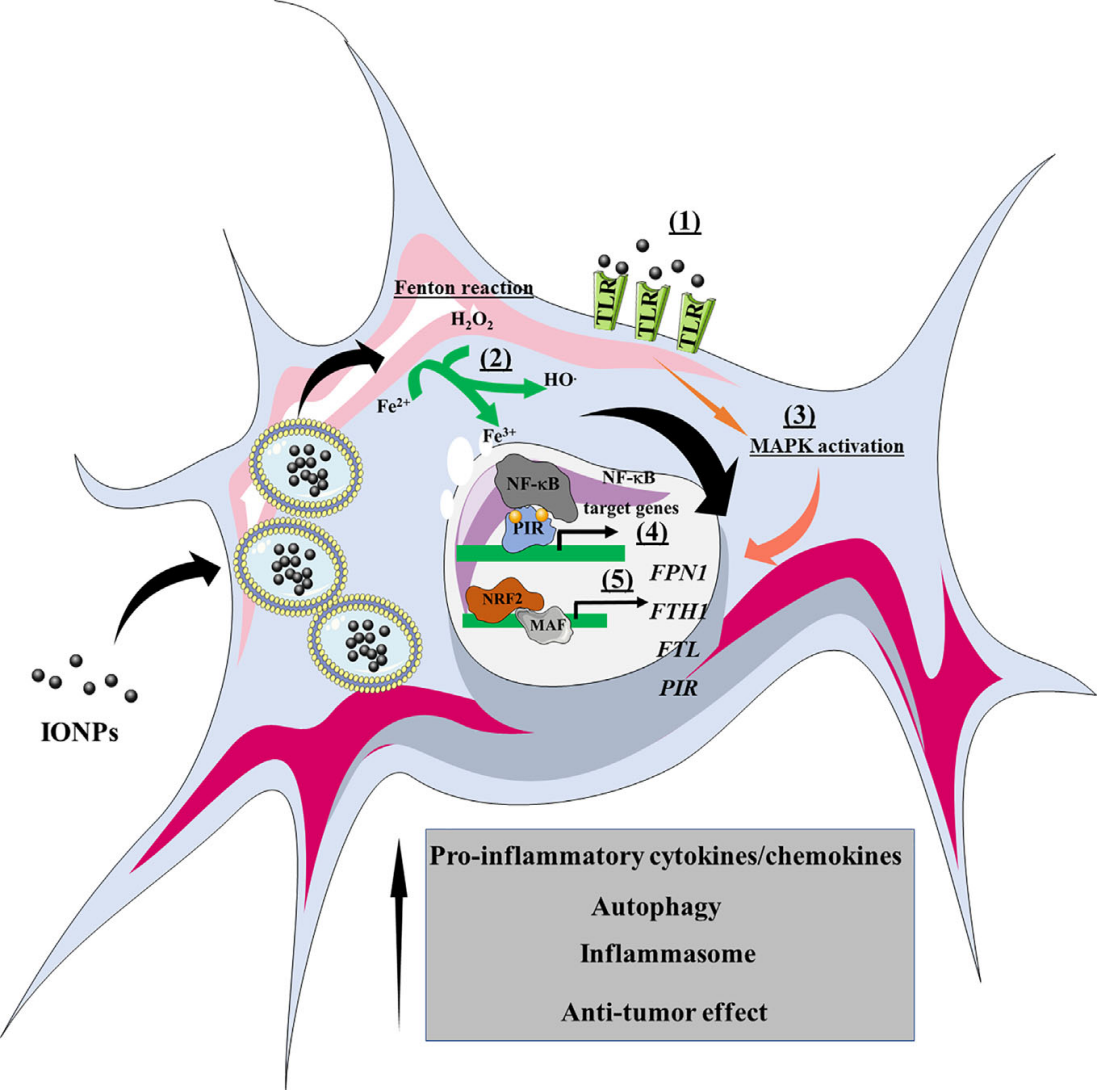
Overview of the effects of IONPs on macrophage polarization. The IONPs can interact with cell surface receptors such as TLRs (1), leading to activation of the MAPK signaling pathway. Once internalized by macrophages, the IONPs are enclosed within endolysosomes where they are biodegraded. Consequently, atomic iron is released into the cytoplasm, where it engages the Fenton reaction and produces ROS (2). As a result, transcriptional reprogramming is triggered, such as that involving NF-κB (e.g., cytokines, chemokines) and NRF2 target genes (e.g., iron metabolism). NRF2, nuclear factor (erythroid-derived 2)-like 2; PIR, Pirin; *FPN1*, ferroportin-1*; FTH1*, ferritin heavy chain; *FTL*, ferritin light chain; MAPK, mitogen-activated protein kinases; MAF, musculoaponeurotic fibrosarcoma.

Importantly, each macrophage phenotype expresses different factors involved in iron metabolism, reflected in their distinct iron sensitivity (121). For instance, M2-polarized THP1 macrophages internalize significantly more IONPs than M1-polarized and M0 macrophages, leading to a higher T1 signal in M2 macrophages and a higher T2^*^ signal in M0 macrophages (122). In turn, internalized IONPs could also exert effects on polarization and iron metabolism. Indeed, our group demonstrated that DMSA-, APS-, and aminodextran-coated IONPs shifted iron metabolism towards an iron-sequestering status in M2-like macrophages (103). In the light of the above, we can propose a general overview of the events induced by IONPs that precipitates macrophage activation (**Figure 4**).

IONPs have also been used to track microglia and assayed as a potential nanocarrier in brain tumors. Microglia are highly phagocytic cells found entirely in the central nervous system (CNS) where they protect the nervous tissue from debris and damaged CNS structures and from viruses, microorganisms, and tumors (123–126). Therefore, like macrophages, microglia can phagocytose IONPs and react to them. In this sense, Wu HY et al. found that the carboxydextran-coated IONP (Resovist™) counteract the LPS-induced microglia activation by directly decrease IL-1β secretion (127), suggesting IONPs can protect CNS from an exacerbated inflammation. However, other reports pinpoint the involvement of IONPs in recruiting and activating microglia in CNS structures such as the olfactory bulb, hippocampus, and striatum. Indeed, Wang Y et al. found that Fe_2_O_3_ IONPs administered intranasally promote the recruitment of microglia into the above CNS structures and induced microglia activation and proliferation, with ROS and nitric oxide (NO) production, as a possible defense mechanism against foreign particulates (128). Thus, IONPs appear to change CNS immunological microenvironment toward an inflammatory or anti-inflammatory phenotype, highlighting the need to comprehend these effects in the context of brain tumors.

## Therapeutic Iron Oxide Nanoparticle-Enabled Modulation of TIME

We have discussed the activation of macrophages by IONPs and the molecular mechanisms mediating these effects. Considering the intrinsic biological activity of IONPs on macrophages, their application in therapeutic and prophylactic vaccination schemes has emerged as an attractive therapeutic approach to treat cancer. This approach relies on the possibility of combining the carrier capacity of IONPs with their by-stander activation of macrophages within the TIME. A general overview of IONP-based vaccine designs highlights the use of IONPs as an antigen carrier (primarily associated with the tumor cells)with the possible addition of adjuvant and/or a targeting moiety.

The use of IONPs as an antigen carrier in a vaccination schedule takes advantage of the intrinsic capacity of the IONPs to drive macrophages or DCs towards a pro-inflammatory phenotype. Consequently, antigen internalization, intracellular processing, and restricted major histocompatibility complex (MHC) presentation to T cells within an inflammatory microenvironment will elicit a robust immune response against the antigen-expressing tumor cells. A simple vaccine formulation has been tested by loading ovalbumin (OVA) onto IONPs, demonstrating that this formulation could activate bone marrow-derived dendritic cells (BMDCs) and RAW 264.7 macrophages. However, the most exciting finding was that prophylactic or therapeutic injection of three doses of this preparation delayed OVA-expressing B16 tumor cell growth.

Interestingly, OVA-coated IONPs effectively prevented lung metastasis from OVA-expressing cells (129). Likewise, the sole conjugation of OVA alone with IONPs was sufficient to elicit potent DC and macrophage activation, and to reduce the OVA-expressing CT26 tumor burden *in vivo* (130). This anti-tumor effect appeared to be mediated by the induction of pro-inflammatory cytokines like IL-6, TNF-α, and IFN-γ.

Other studies have addressed the potential of the IONPs as carriers of tumor-associated antigens in vaccine designs. For example, the administration of self-assembled MUC1 lipo(glycol)peptide-coated IONPs elicited a strong antibody response, prompting an antibody profile able to recognize the MUC1-expressing tumor cell line, MCF7 (131). In this scenario, the anti-tumor effect seems more likely to be related to the enhanced activation of plasma B cells due to the high number of lipo(glycol)peptide copies presented on the IONP surface. However, we cannot rule out a direct effect on macrophages or DCs.

It is desirable that macrophage-based anti-tumor therapy induces naive macrophages to adopt a M1 phenotype and that it switches the resident M2 program into a M1 phenotype, ensuring a pro-inflammatory and anti-tumor TIME. It was seen that hyaluronic acid-modified IONPs or bare IONPs trigger the production of ROS and pro-inflammatory cytokines (132). Consequently, IONP-treated macrophages exerted an anti-tumor effect on the murine 4T1 breast-tumor cell line in a cell contact-independent manner, inducing active caspase 3 and inhibiting cell proliferation. Notably, hyaluronic acid-modified IONPs induced M1 macrophages resistant to M2-inducing factors and M2-to-M1 macrophage reversion (132). IONP intracellular degradation also increases the labile iron pool, providing another element that can modulate the TIME. It was shown that red blood cells (RBCs) were responsible for the presence of iron-loaded macrophages nesting in the invasive margins of non-small lung cell tumors, which were in turn correlated with a smaller tumor size (133). Indeed, hemolytic RBCs triggered TAM polarization toward a M1-like phenotype, as evident by the expression of M1 marker transcripts (*Il6, Nos2*, and *Tnfa*) and their increased anti-tumor activity (133). More importantly, IONPs injected intravenously in Lewis lung carcinoma (LLC)-bearing mice accumulated within F4/80 macrophages and reduced tumor growth, indicating that these IONPs have a similar effect reverting M2 macrophages to a M1 phenotype (133).

Advantages have also been reported when a combination of antigen-coated IONPs and adjuvant-coated IONPs is used therapeutically. While IONPs were initially used as antigen carriers, adjuvant and nanoparticle association enhanced the adjuvant effect on the respective signaling pathway. Indeed, co-delivery of polyIC-R837@mPEG-PL-OA-IONPs (as TLR3-7 agonists) and OVA@mPEG-PL-OA-IONPs (as antigen) delayed tumor growth in OVA-expressing B16-bearing animals and led to tumor-free survival in some individuals, probably through an enhanced agonist effect on TLR signaling. The increase in the ferroptosis process induced by IONP-derived iron further promoted an antitumoral TME, indicating that the IONPs provide not only transport but also an intrinsic potential to change the TME toward an anti-tumor phenotype (134). **Table 4** summarizes the most recent approaches using IONPs in anti-tumor vaccination regimens.

**Table 4:** Use of IONPs in vaccine formulation.

Nano-formulation	Tumor model	Effects
Ovalbumin@Fe_3_O_4_ (129)	Murine melanoma OVA-expressing B16	Bone marrow-derived DC maturation
		Therapeutic and prophylactic inhibition of tumor growth
		Therapeutic and prophylactic attenuation of lung metastasis
OVA@Fe_3_O_4_ (130)	Murine colon carcinoma OVA-expressing CT26	Murine dendritic cell (DC2.4) and macrophage (RAW 264.7) activation (increased IL-6, TNF-α and IFN-γ)
		Therapeutic anti-tumor effect (reduced CT26 tumor growth and increased serum IL-6, TNF-α and IFN-γ)
OVA/CpG/anti-DEC205 Ab@Fe_2_O_3_ (135)	Murine melanoma OVA-expressing B16	*In vivo* targeting of CD8^+^ DCs
		*In vivo* B16 tumor arrest
Hsp70@SPION (136)	Murine C6 glioma	DCs, tumor lysate and Hsp70@SPION co-treatment arrests glioma tumor growth
MUC1 Lipoglycopeptide@SPION (131)	MCF7	Multivalent engagement of antibody-producing B cells.
		Generation of a strong antibody response *in vivo*.
		Tumor cell recognition and cell death by immunized sera.
Co-delivery of micellar OVA@phospholipid-PEG-IONP & LOS@ phospholipid-PEG-IONP (137)	OVA-expressing B16-F10	Increased IL-6 and reduced LOS cytotoxicity
		Prophylactic anti-tumor effect & synergetic effect with PD-L1 inhibitor
Co-delivery of polyIC-R837@mZnSPION & OVA@mZnSPION (93)	OVA-expressing B16-F10	Micellar ZnSPION enhances TLR3/7 agonist effects
		Prophylactic and therapeutic anti-tumor effect & synergetic effect with PD-L1 inhibitor
Co-delivery of polyIC-R837@mPEG-PL-OA-IONP & OVA@mPEG-PL-OA-IONP (134)	OVA-expressing B16-F10	Enhances TLR agonist effects on DCs
		Improves the tumor-free rate over time
		Synergistic effects with immunostimulatory antibodies (anti-OX40 & anti-PD-L1)

## Conclusions

IONPs have been studied intensively in recent decades to exploit their magnetic and surface chemical features. However, only recently has attention been drawn to their intrinsic immunomodulatory properties, especially their effects on macrophages. These effects are particularly important in the context of cancer immunotherapy as IONPs can provide an efficient vehicle for antigen delivery and elicit a potent immune response, reprogramming TAMs toward an immunogenic phenotype. Two main molecular mechanisms can explain the intrinsic immunomodulatory effect of IONPs: 1) the production of ROS and consequently, the modulation of redox-sensitive signaling pathways; and 2) the direct engagement and activation of immune response-related receptors, such as TLRs, inducing transcriptional reprogramming in macrophages. The use of IONPs can provide a reliable platform to reprogram the typical M2-TAM phenotype toward a pro-immunogenic phenotype, synergizing with currently used immunotherapies like checkpoint inhibitors to mount a potent anti-tumor immune response both locally and systemically.

## Author Contributions 

VM-A, JR, and DB conceived and designed the review. VM-A and JR wrote sections of the manuscript, and prepared the figures and tables. DB coordinated, critically revised, modified and completed the manuscript. All authors contributed to the article and approved the submitted version.

## Funding

VM-A is a post-doctoral scholar working under a Juan de la Cierva-Incorporación Contract (IJCI-2017-31447) from the Spanish Ministry of Science and Innovation. The European Commission-funded VetBioNet INFRAIA-731014 project supports JR. This work was supported in part by grants from the Spanish Ministry of Science and Innovation (SAF-2017-82223-R and PID-2020-112685RB-100 to DB). DFB group is part of the Network “Nanotechnology in Translational Hyperthermia” (HIPERNANO, RED2018-102626-T) supported by the Spanish Ministry of Science and Innovation.

## Conflict of Interest

The authors declare that their research was conducted in the absence of any commercial or financial relationships that could be construed as a potential conflict of interest.
